# Predicting deterioration in dengue using a low cost wearable for continuous clinical monitoring

**DOI:** 10.1038/s41746-024-01304-4

**Published:** 2024-11-02

**Authors:** Damien Keng Ming, John Daniels, Ho Quang Chanh, Stefan Karolcik, Bernard Hernandez, Vasileios Manginas, Van Hao Nguyen, Quang Huy Nguyen, Tu Qui Phan, Thi Hue Tai Luong, Huynh Trung Trieu, Alison Helen Holmes, Vinh Tho Phan, Pantelis Georgiou, Sophie Yacoub, On behalf of the VITAL consortium

**Affiliations:** 1 Centre for Antimicrobial Optimisation, Imperial College London, London, UK; 2 Centre for Bio-Inspired Technology, Imperial College London, London, UK; 3 Oxford University Clinical Research Unit, Ho Chi Minh City, Vietnam; 4 Hospital for Tropical Diseases, Ho Chi Minh City, Vietnam; 5 Department of Global Health and Infectious Diseases, University of Liverpool, Liverpool, UK; 6 Centre for Tropical Medicine and Global Health, Nuffield Department of Medicine, University of Oxford, Oxford, UK

**Keywords:** Risk factors, Predictive markers, Viral infection

## Abstract

Close vital signs monitoring is crucial for the clinical management of patients with dengue. We investigated performance of a non-invasive wearable utilising photoplethysmography (PPG), to provide real-time risk prediction in hospitalised individuals. We performed a prospective observational clinical study in Vietnam between January 2020 and October 2022: 153 patients were included in analyses, providing 1353 h of PPG data. Using a multi-modal transformer approach, 10-min PPG waveform segments and basic clinical data (age, sex, clinical features on admission) were used as features to continuously forecast clinical state 2 h ahead. Prediction of low-risk states (17,939/80,843; 22.1%), defined by NEWS2 and mSOFA < 6, was associated with an area under the precision-recall curve of 0.67 and an area under the receiver operator curve of 0.83. Implementation of such interventions could provide cost-effective triage and clinical care in dengue, offering opportunities for safe ambulatory patient management.

## Introduction

Dengue exerts a significant global healthcare burden. A minority of symptomatic individuals (5-10%) develop severe disease characterised by a vascular leakage syndrome which can then lead to cardiovascular decompensation and death^1^. Close monitoring of clinical status and vital signs allows for the identification of patients at an increased risk of progression. The interpretation of these clinical features in turn guides delivery of supportive therapies, including fluid resuscitation and organ support measures^2^. With the growing incidence and expansion of at-risk regions as a result of climate change^3^, dengue represents a major challenge faced by healthcare systems worldwide.

Patients with dengue infection present with a non-specific febrile illness which typically evolve over 3–4 days. Existing clinical tools, such those published by the WHO, play a crucial role in standardised pre-hospital triage and are commonly used as criteria for hospitalisation. However, such evaluations performed at a single point of encounter are at risk of failing to capture the dynamic nature of this disease^4^. Regular, and repeated patient monitoring coupled with contemporaneous assessments allow for better clinical prioritisation, improving patient outcomes as well as healthcare effectiveness^5^. Within many low- and middle-income countries where dengue is endemic, such in-hospital patient monitoring can prove challenging for a range of reasons, including lack of functional equipment, gaps in training and shortages in healthcare staff^6^.

Non-invasive wearable devices could offer benefits through providing cost-effective individualised monitoring in healthcare^7^. Many wearables utilise photoplethysmography (PPG) as the primary sensing modality due to low power requirements and safety^8^. Emitted light from the device is distorted by passage of the arterial waveform through underlying skin tissue, and this signal is captured as a continuous waveform. Many PPG devices are market-approved in their use as pulse oximetry, and there is growing evidence that their deployment can prove clinically-effective, such as for the delivery of safe surgery in low income settings^9^, or remote patient monitoring in COVID-19^10^.

Within dengue specifically there is support that PPG signals are predictive of shock in patients with severe disease admitted to the intensive care^11^; the PPG waveform is closely related to blood pressure, respiratory rate and fluid-volume status^12^. Wearables deployed appropriately in general ward settings could support a real-time system and assist in clinical prioritisation of patients or guide fluid therapy. Coupled with data linkage, the timely identification of individuals at high risk of disease progression, or conversely patients who are stable and can be safely de-escalated could improve resilience in healthcare systems and translate into better clinical outcomes.

We performed a prospective observational study in patients admitted to hospital in Vietnam for the management of dengue, with the aim of investigating the relationship between continuous PPG monitoring, physiological derangement and illness severity. The objective is the development of a real-time clinical decision support framework which could be implemented in such healthcare settings to support early triage and management of dengue.

## Results

Between 5th January 2020 and 6th October 2022, 906 patients were screened and assessed for eligibility. For those patients deemed unsuitable for recruitment because of clinical reasons (*n* = 241), the majority were excluded because of a stated contraindication to the use of hardware device (*n* = 223) which include delirium and agitation, deformity of limb, known allergy to device component and pregnancy. Patients who were discharged to die at home (*n* = 5) and presenting late in the recovery phase (*n* = 13) were also excluded.

We enroled 250 patients into the study and included 153 (61.2%) patients in the analysis based on PPG data quality criteria and data availability. A median duration of 10.2 (IQR 4.0–11.9) hours of continuous PPG signal were available for each patient, and the total duration of PPG data in final analyses was 1353 h.

The median age of the analysis cohort (*n* = 153) was 29 (IQR 21–34) years old and 89 (58.2%) were female. In total 96/152 (63.2%) patients were diagnosed with severe dengue on their entire hospital admission, and 16/56 (28.6%) patients who were admitted without severe dengue, subsequently developed dengue shock syndrome during the course of their admission.

The median NEWS2 score over the initial 24-h admission period was 2 (IQR 2–4) and the median mSOFA score was 6 (IQR 3–7). For each patient, the period of continuous monitoring was divided into 10-min rolling window segments which advanced by 1-min increments. The number of segments classified as low risk for the whole cohort was 22.2% (17,939/80,843).

**Fig. 1:**
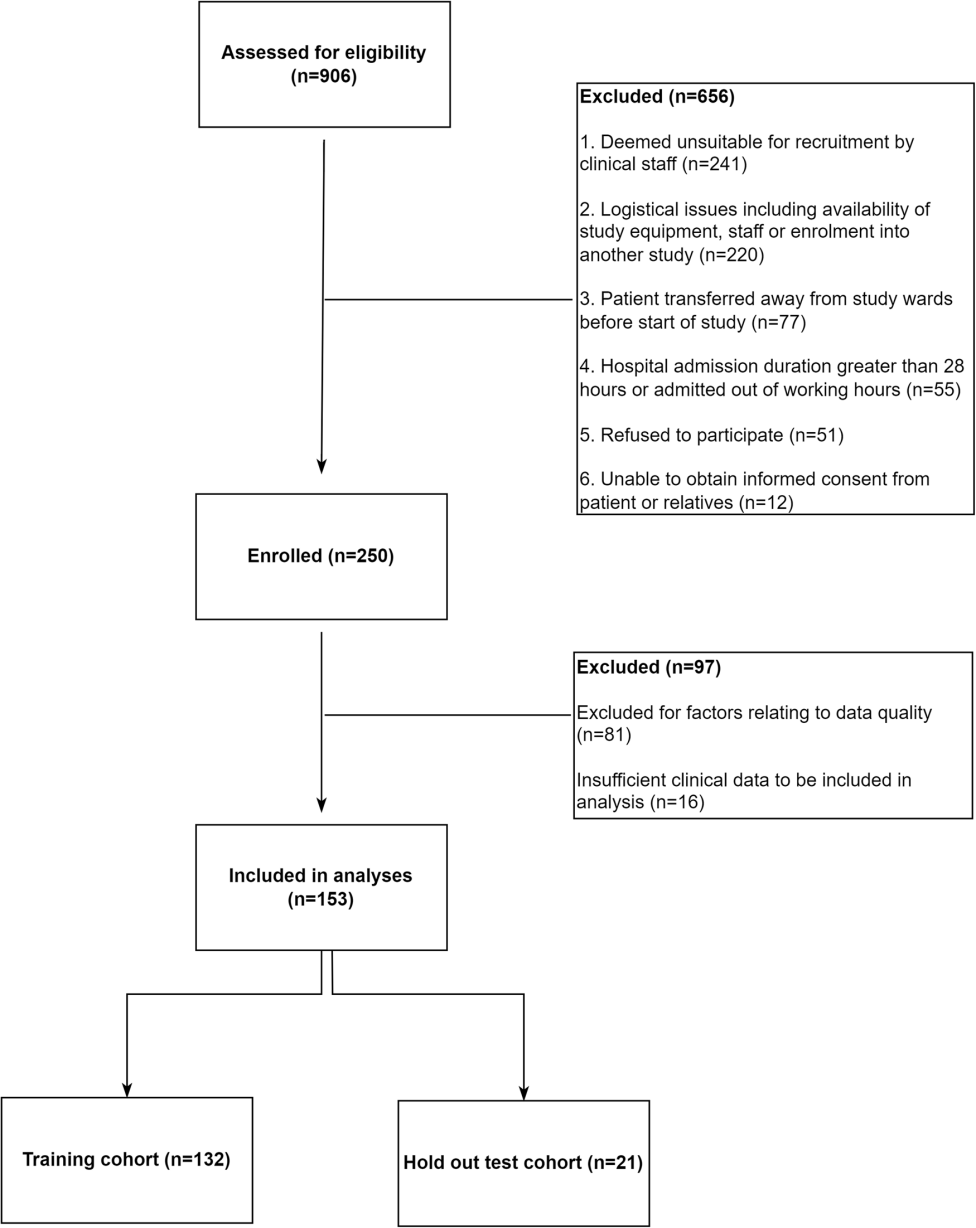
CONSORT diagram of patient recruitment and flow. The figure shows the outline for the recruitment process in which *n* = 250 participants were enroled, and *n* = 153 included in the analyses. The cohort was randomly split into a training (*n* = 132) and hold out test (*n* = 21) cohort.

The baseline characteristics of the whole study cohort and distribution of NEWS2 and mSOFA scores by train/test set is shown in Table 1, a description of the baseline characteristics of the excluded patients is shown in supplementary table 1 and recruitment flow is shown in Fig. 1.

**Table 1:** Baseline characteristics of the patients

		Overall	Training set	Hold out test set
n		153	132	21
Age in years, median [Q1, Q3]		29.0 [21.0, 35.0]	29.0 [21.0,35.0]	30.0 [20.0, 33.0]
Gender, median [Q1, Q3]	Female	89 (58.2)	73 (55.3)	16 (76.2)
	Male	64 (41.8)	59 (44.7)	5 (23.8)
Weight in kg, median [Q1, Q3]		60.0 [52.0, 72.0]	61.5 [53.0, 74.0]	54.0 [50.0, 60.2]
Pulse (bpm), median [Q1, Q3]		88.0 [80.0, 100.0]	88.0 [80.0, 100.0]	86.0 [79.0, 96.0]
Systolic blood pressure, mmHg, median [Q1, Q3]		110.0 [100.0, 120.0]	110.0 [100.0, 120.0]	110.0 [100.0, 116.7]
Diastolic blood pressure mmHg, median [Q1, Q3]		70.0 [70.0, 80.0]	70.0 [70.0, 80.0]	70.0 [70.0, 80.0]
Respiratory rate, breaths per minute, median [Q1, Q3]		20.0 [20.0, 22.0]	20.0 [20.0, 22.0]	20.0 [20.0, 22.0]
Oxygen saturation percent, mean (SD)		96.4 (8.5)	96.3 (9.1)	97.4 (1.4)
Body temperature in Celsius, median [Q1, Q3]		37.0 [37.0, 37.5]	37.0 [37.0, 37.5]	37.2 [37.0, 37.5]
Haematocrit percent, median [Q1, Q3]		44.8 [40.8, 48.6]	44.7 [40.7, 48.5]	44.8 [41.0, 49.7]
NEWS2 score, *n* (%)	1–4	2844 (83.3)	2371 (81.8)	473 (91.7)
	5–6	421 (12.3)	379 (13.1)	42 (8.1)
	7+	148 (4.3)	147 (5.1)	1 (0.2)
mSOFA score, *n* (%)	1–6	1690 (45.3)	1378 (42.7)	312 (62.7)
	6-7	1913 (51.3)	1727 (53.5)	186 (37.3)
	8+	124 (3.3)	124 (3.8)	
Clinical risk score, *n* (%)	Low risk	847 (22.1)	624 (18.9)	223 (42.5)
	Higher risk	2978 (77.9)	2676 (81.1)	302 (57.5)

Baseline characteristics of patients (*n* = 153) included in analysis and model development, separated by training (*n* = 132) and hold out test set (*n* = 21).

### Predictive models

#### Dengue shock syndrome

Models predicting dengue shock syndrome alone over a 2-h horizon were developed and evaluated. In total, 8394/80,843 (10.4%) of study period segments were classified as dengue shock. All optimised models had similar performance in 5-fold cross validation. The STF Multi-task learning (MTL) approach achieved a mean area under the precision-recall curve (AUPRC) of 0.15 (standard deviation, SD ± 0.03), AUROC of 0.72 (SD ± 0.03), F1 score of 0.28 (SD ± 0.02), precision of 0.16 (SD ± 0.01) and recall 0.97 (SD ± 0.02) against the independent hold out test set for each of the models trained in 5-fold cross validation. Full results are shown in Table 2.

**Table 2:** Performance of different models on predicting dengue re-shock over a 2-h prediction horizon

Metric	STF (MTL)	STF (STL)	CNN-LSTM	CNN
AUPRC	0.15 ± 0.03	0.15 ± 0.03	0.10 ± 0.04	0.08 ± 0.01
AUROC	0.72 ± 0.03	0.72 ± 0.04	0.53 ± 0.12	0.53 ± 0.05
F1	0.28 ± 0.02	0.27 ± 0.03	0.14 ± 0.08	0.12 ± 0.04
Precision	0.16 ± 0.01	0.16 ± 0.02	0.08 ± 0.05	0.08 ± 0.02
Recall	0.97 ± 0.02	0.90 ± 0.13	0.33 ± 0.20	0.28 ± 0.10

Mean values from hold out set testing for each of the models in 5-fold cross validation are presented.

*AUPRC* area under the precision-recall curve, *STF* spatio-temporal fusion transformer, *MTL* multi-task learning, *STL* single-task learning, *CNN* convolutional neural network, *LSTM* long short-term memory.

#### Prediction of patient illness severity

Models predicting illness severity score based on the composite NEWS2 / mSOFA and clinical criteria were associated with higher discrimination. Models based on PPG data alone to predict outcomes were associated with a mean AUPRC of 0.43 (SD ± 0.07), area under the receiver operator curve (AUROC) of 0.58 (SD ± 0.07), F1 score of 0.54 (SD ± 0.07), precision of 0.45 (SD ± 0.04) and recall of 0.68 (SD ± 0.14) when each of the models from cross validation were evaluated against the hold out test set.

Where a multi-modal approach was taken to incorporate clinical information to PPG, MTL was associated with the highest average performance when evaluated against the hold out test set. Models were associated with a AUPRC of 0.67 (SD ± 0.12), AUROC of 0.83 (SD ± 0.07), F1 of 0.78 (SD ± 0.05), precision of 0.69 (SD ± 0.02), recall of 0.95 (SD ± 0.03). Multi-modal signal task learning approaches still had greater performance over models which utilise PPG signal alone.

Sensitivity analyses using clinical features alone to predict clinical state were done using time-invariant models to predict patient outcome during the admission period, but these showed poorer discrimination compared with those incorporating PPG data.

Full results are shown in Table 3.

**Table 3:** Mean performances over 5-fold cross validation

Metric	STF (MTL)	STF (STL)
AUPRC	0.67 ± 0.12	0.58 ± 0.06
AUROC	0.83 ± 0.07	0.71 ± 0.08
F1	0.78 ± 0.05	0.68 ± 0.08
Precision	0.69 ± 0.02	0.60 ± 0.08
Recall	0.95 ± 0.03	0.82 ± 0.17

Performances of spatio-temporal fusion transformer (STF) models utilising multi-task learning (MTL) and single-task learning (STL) on predicting patient illness severity over a 2-h horizon.

## Discussion

In this study we utilised a low-cost, non-invasive PPG wearable for the continuous monitoring of a large cohort of patients hospitalised with dengue in Vietnam. Deep learning models allowed for risk stratification to take place in real time, and predict patient derangements during early admission. Two robust assessment scores: the NEWS2 and mSOFA were combined and used as a proxy for patient illness severity. A multi-task approach integrating both basic clinical information with PPG waveform data was associated with better predictive performance: the optimised STF model predicted individual patient risk 2 h ahead and with an AUROC of 0.83 and AUPRC of 0.67. Use of MTL to exploit similarities between two related outcomes also improved the quality of predictions. However, the prediction of dengue shock syndrome was associated with significantly lower performances. As DSS is a clinical diagnosis, it is possible the label may be subject to a treatment or ground truth bias—patients might have their physiology normalised quickly through treatment, or remain underdiagnosed.

Wearable devices are inherently low powered and can be miniaturised. The PPG device used in this study cost ~$100 USD each and could be reused several times. The clinical utility and cost-effectiveness of such interventions is likely increased in resource-restricted settings and during periods of high dengue caseload, where frequent clinical observations can be difficult. We have previously developed machine learning-based scoring system for dengue management^13^. Coupled with network connectivity^14^, such wearables could provide important information to healthcare staff in real time to augment clinical prioritisation. Given established relationships between PPG and haemodynamic status, it is possible that information relating to fluid treatment response can be derived and guide treatment. The cost, maintenance of new medical equipment and delivery of critical care in resource restricted healthcare settings remains a major issue^15^, and as such medical wearables could fulfil an important role.

Although the study was done in a hospital setting, the majority of patients with dengue in Vietnam are managed on an outpatient basis. We speculate that some of the early changes in PPG would be useful to predict clinical deterioration in the community. However, it would be crucial for future studies to recruit participants managed in the community, and with this there could be future scope to utilise wearables for remote patient monitoring and syndromic monitoring^16,17^. Coupled with other data and sensor modalities including biosensing^18^, accelerometery and self-entered information, multi-modal approaches and the integration of diverse data types will be likely be increasingly important over time.

Strengths of the study include the prospective pragmatic study design, large cohort of dengue patients recruited and use of state-of-the-art deep learning and model development. We adopted a conservative clinical definition of patient illness severity in order to reliably identify patients which at lowest risk of deterioration, given the importance of avoiding false negative results. It would be important such thresholds undergo appropriate calibration in prospective studies to achieve optimal trade-off between false negatives and performance.

Limitations include the low granularity of vital signs collection, which was done typically every 6 h on ward settings, and might have contributed to a ground truth bias. Although the NEWS2 and mSOFA score correlate with clinical state across different conditions, the specific thresholds and utility specifically for the management of dengue has yet to be determined. Movement artefact and noise also hindered signal quality in PPG—it is possible reduced pulse waveform amplitudes and decreased peripheral perfusion would affect the capture of PPG signals from the wrist and fingertip—specific hardware adaptations and filtering might improve data quality in the future.

This was a pragmatic observational study in a middle-income healthcare setting—we found that the attending clinical team expressed uncertainty regarding the impact of an additional wearable device to routine patient care, particularly in the critical care settings where concurrent use of other monitoring devices was common. On average 10.2 h out of 24 (43%) of data were deemed of sufficient quality for analysis which resulted the exclusion of a number of patients in analysis (*n* = 97; 38.8%). The hardware used also had a finite battery life (18 h of continuous monitoring on average) which meant interruptions to continuous monitoring beyond this duration.

Regarding the relatively modest discrimination ability of the models to predict dengue shock syndrome, one explanation for results relates to the class imbalance of the data. For each individual, the period of dengue shock (and associated PPG data) is relatively infrequent compared to periods without dengue shock, and this might have hindered model training. The clinical priority is to minimise exposure to shock states through judicious resuscitation. Given this was a study done in LMIC settings, labelled onset of dengue shock can also be subject to variation particularly if vital signs monitoring is sparse although this is minimised through the study protocol.

From qualitative feedback, the relatively large form factor of the existing PPG wearable used in our study also hindered patient adherence and comfort in this setting (data not shown). These human factors are likely to influence the nature of data collection. Work is currently underway in the development of appropriate miniaturised hardware for dengue, particularly the addition of sensors to evaluate fluid status through haematocrit measurement^19^. An important facet of treatment is the identification of fluid overload states to indicate the adequacy, and guide fluid resuscitation. Evaluation of these additional endpoints such as correlation with presence of clinical plasma leakage would be add value.

In conclusion, integration of PPG signals with clinical information through a multi-modal, multi-task deep learning approach predicted patient illness severity in real time for patients hospitalised with dengue. As a proof of concept, our findings are likely applicable to other clinical states resulting result in haemodynamic compromise, such as trauma, sepsis or perioperative conditions. It is important that a robust bioengineering approach for appropriate hardware design, secure data integration and clinical implementation studies examining impact in our resource setting^20^ is adopted.

## Methods

The aims of the study were to utilise continuous PPG signals captured through a non-invasive wearable to develop, and evaluate deep learning models which provide real-time classification of patient illness severity and predict clinical outcomes for adult and paediatric patients hospitalised with dengue in Southern Vietnam during early admission (<24 h). Findings are reported in line with the STROBE guidelines^21^.

### Setting

This was a prospective observational study performed at the Hospital for Tropical Diseases (HTD), a tertiary hospital in Ho Chi Minh City, Vietnam. The hospital provides specialist infectious diseases services to Southern Vietnam and manages ~30,000 patients with dengue each year. Patients with an acute febrile illness self-present to the outpatient or emergency department, or are transferred from regional healthcare centres for further evaluation and management.

### Participants

The inclusion criteria for the study are:

- Age 8 or older admitted to hospital, *and*
- Clinical diagnosis of dengue, *and*
- Hospitalisation less than 28 h at time of enrolment.

Exclusion criteria are:

- Lack of informed consent /assent from patient (or from relatives in children), *or*
- Clinical reasons at discretion of treating staff.

Settings for patient recruitment include the Emergency Department, Paediatric and Adult intensive care units and general wards to increase the diversity of presentations. Dengue diagnosis was defined as a clinically compatible febrile syndrome with a positive dengue NS1 assay. These definitions are clinically pragmatic and consistent with guidelines published by the national Ministry of Health in Vietnam.

### Enrolment and study processes

Informed consent was obtained by attending clinicians in accordance with Good Clinical Practice. For children aged 12 or older, assent was obtained in addition to consent from the parent or guardian. Clinical information including demographics, presenting symptoms, examination findings and vital signs were recorded on enrolment. Specifically, blood pressure, pulse rate, respiratory rate, tympanic temperature and oxygen saturation were captured on study case report forms. Additional administered treatment and vital signs during the 24-h study period performed as part of standard clinical care were recorded; with patients typically undergoing observations every 1–6 h depending on clinical need.

PPG data recording was done with a battery-operated wrist wearable containing a transmissive finger probe (SmartCare Analytics, Oxford, UK). This device continuously captured timestamped PPG signals from the patient using red and infra-red wavelengths at 100 Hz, and stored data on the internal memory which was downloaded after end of the recording period. Participants wore the device on their finger for up to 24 h after enrolment. A description of the device and its appearance is presented in supplementary fig. 1.

### Sample size

The target sample size for the study was 250, based on pragmatic factors including feasibility of clinical recruitment during one dengue season, likely participant drop-out and data quality issues.

### Measurements and definition of outcomes

Patients attending the HTD are assessed according to the national Ministry of Health guidelines and admitted based an increased risk of disease progression (such as presence of warning signs) or presentations consistent with severe disease. Those who do not have any clinical or social risk factors tend to be managed as outpatients.

The primary outcome for the model was the prediction onset of clinical shock (dengue shock syndrome or recurrent shock) as a discrete event 2-h ahead of time, according to the WHO Dengue Case Classification 2009 definitions using a 10-min segment of PPG recording.

Dengue shock syndrome is the most common clinical manifestation of severe dengue and is defined as a pulse pressure equal to or less than 20 mmHg, or low blood pressure (BP) for age, with clinical signs of reduced peripheral perfusion. Where a repeated episode of shock occurs after resolution of the initial shock during the same admission, this was defined as recurrent shock according to the criteria above.

Secondary outcomes were patient illness severity during the initial 24-h period of hospitalisation. Each patient was assigned an illness severity score for every hour initial admission in parallel with continuous PPG monitoring. We used two complementary score-based measurements to quantify the degree of physiological derangement and illness severity during this period:

i. The NEWS2 score^22^ is based on vital signs measurements and aggregates derangements in six accessible vital signs parameters: respiratory rate, pulse rate, oxygen saturation, systolic blood pressure, level of consciousness and body temperature. There exists a close relationship between NEWS2 score and all-cause mortality across different conditions^23^.
ii. The dengue-specific modified Sequential Organ Failure Assessment score (mSOFA) was calculated. The score aims to account for limitations in existing approaches to assessing disease severity after hospitalisation. The score has been validated for in a large cohort of dengue patients for our setting and is predictive of clinically-relevant outcomes including requirement for organ support, length of stay and mortality^24^.

A binary outcome was derived based on the physiological derangement in order to identify the patients as low risk of severe disease and do not require immediate intervention. A low clinical risk score was defined as:

i. NEWS2 score less than 6, *and*
ii. mSOFA score less than 6 *and*
iii. the absence of shock or significant bleeding on clinical assessment

We chose a NEWS2 score less than 6 based on the threshold recommendations from the Royal College of Physicians for urgent clinical intervention^22^, and the mSOFA score less than 6 to include patients in the lower 25^th^ percentile of risk from the original derivation study^24^.

### Data partitioning

In order to better evaluate the generalisation of the model for this task, we partitioned our dataset into the usual train, validation and test datasets. At the first step, we first randomly partition the data at a 7:1 ratio to create a hold-out test set with a unique set of patients while maintaining the distribution of classes. Similarly, the remaining data is then split through a stratified 5-fold cross validation to create training and validation sets where the patients are unique to each set while maintaining the distribution of samples between the datasets. The split is implemented using the Stratified K-Fold from the Python scikit-learn package. For each fold, the optimised model was then evaluated against the independent hold out test set to report summary performances. The dataset was randomly split into a training (*n* = 132) and independent hold out test set (*n* = 21) with the latter used exclusively for model evaluation.

### Model features and approaches

PPG signals were the primary feature used in the study. These were collected continuously during the first 24 h of patient admission to hospital and were processed through a rolling window approach. For each individual the feature set consisted of 10-min segments of PPG from start, which were incremented by 1 min until end of the recording period. PPG segments were discarded if the amount of data with corresponding labels was less than the minimum sequence length used by the model (10 min of PPG). For each 10-min PPG segment, a binary outcome prediction was made of the outcome state which was fixed 2 h ahead. For clinical data where an observation has not been performed, a last observation carried forward strategy was adopted. Signal quality indices used include Matching of multiple systolic wave detection algorithms (M_SQI_) and Zero Crossing approaches adapted for PPG data in our project^25^.

Clinical features which were included in the multi-modal models include age, weight, height, gender and presence or absence of headache, vomiting, diarrhoea, abdominal pain on presentation, as well as presence or absence of hypertension and diabetes mellitus in past medical history.

A range of time-series deep learning approaches were used to capture the time series dependencies in the PPG signal. Models including Spatio-temporal Fusion Transformer (SFT)^26^ with, convolutional neural networks (CNN) with, or without Long Short Term Memory (CNN-LSTM) were employed^27^. MTL^28^ was used in order for the same model to concurrently predict multiple outcomes (i.e. dengue shock and illness severity score) in order to augment training.

Specifically, spectrograms of PPG signal sequences of 10-min durations were generated through Short Time Fourier Transform which were then fed into layers a single frame at a time, with or without the addition of recurrent layers to model temporal change. An attention layer was used to model longer term relationships between data and outcomes and provide some interpretability. Multi-modal approaches incorporate demographic, and clinical information (age, weight, height, presence of co-morbidities, dengue warning signs and presence of shock on hospital admission) with the PPG data were then developed and evaluated.

### Evaluation metrics

The prediction outcomes are binary, and the following metrics were incorporated: AUROC is derived from the area under the curve plotting the true and false positive rate at all thresholds as a metric for discrimination. The AUPRC is derived from precision (true positive/(true positive + false positives)) and recall (true positive/(true positive + false negative)) which can provide a better proxy for discrimination ability where class imbalance exists. The F1 score defined as the harmonic mean of precision and recall provides another assessment of discrimination.

**Fig. 2:**
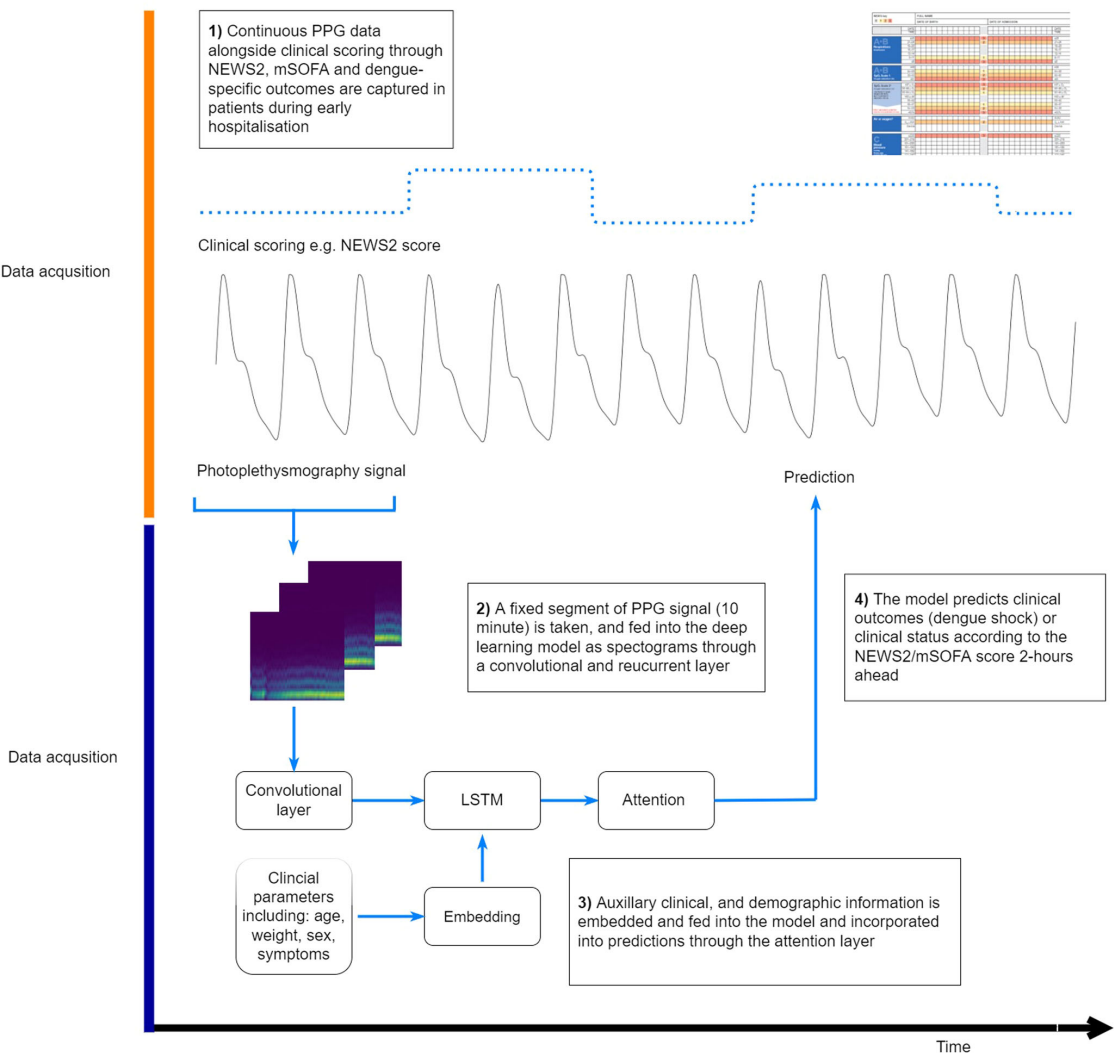
An overview of the model architecture and data in the study. Patients enroled in the study underwent continuous PPG monitoring alongside vital signs measurements to derive a score through NEWS2/mSOFA systems. Each fixed window of PPG was fed into the model which consisted of a convolutional, recurrent and/or attention layers in order to generate predictions 2 h into the future. Auxiliary clinical data were inserted through embedding. A multi-task learning approach was used to improve performances.

The study overview is shown in Fig. 2 and example notebooks are attached.

## Supplementary information

Supplementary material

## Data Availability

Access to the original PPG and clinical data can be applied through the Scientific and Ethical Committee of the Hospital for Tropical Diseases, Ho Chi Minh City.

## Code availability

The code and example notebooks for the data analysis and the optimised model are available at a GitHub repository [hosted at https://github.com/jsmdaniels/VITAL].
