# Supplementary material for: Predicting deterioration in dengue using a low cost wearable for continuous clinical monitoring
Source: NPJ Digit Med. 2024 Nov 2;7:306. doi: 10.1038/s41746-024-01304-4 (PMC11531560; doi:10.1038/s41746-024-01304-4)
Supplement: Supplementary file 1 — Supplementary material [file 41746_2024_1304_MOESM1_ESM.pdf]

Supplementary information to

Continuous non-invasive monitoring in dengue through a low-cost wearable: a prospective observational clinical study.

Table of contents:

Supplementary figure 1: Smartcare analytics PPG device photograph

Supplementary figure 2: NEWS2 and mSOFA distributions of the analysis cohort

Supplementary table 1: Baseline comparison between patients included and excluded from analysis based on signal quality indices

Supplementary figure 3: NEWS2 scoring system

Supplementary table 2: modified SOFA scoring system

Individual authors for the VITAL consortium

Supplementary figure 1: Photograph of the SmartCare Analytics monitor. The device is battery powered with the processing unit worn on the wrist, collecting PPG data at 100Hz for the red 670 nm and infrared 940 nm. The sensor probe used is either wrapped around the finger (reflective PPG) or placed at the pulp of the finger (transmissive PPG). For the majority of patients a transmissive PPG finger probe was used for signal acquisition. Data was downloaded from the device after the monitoring period.

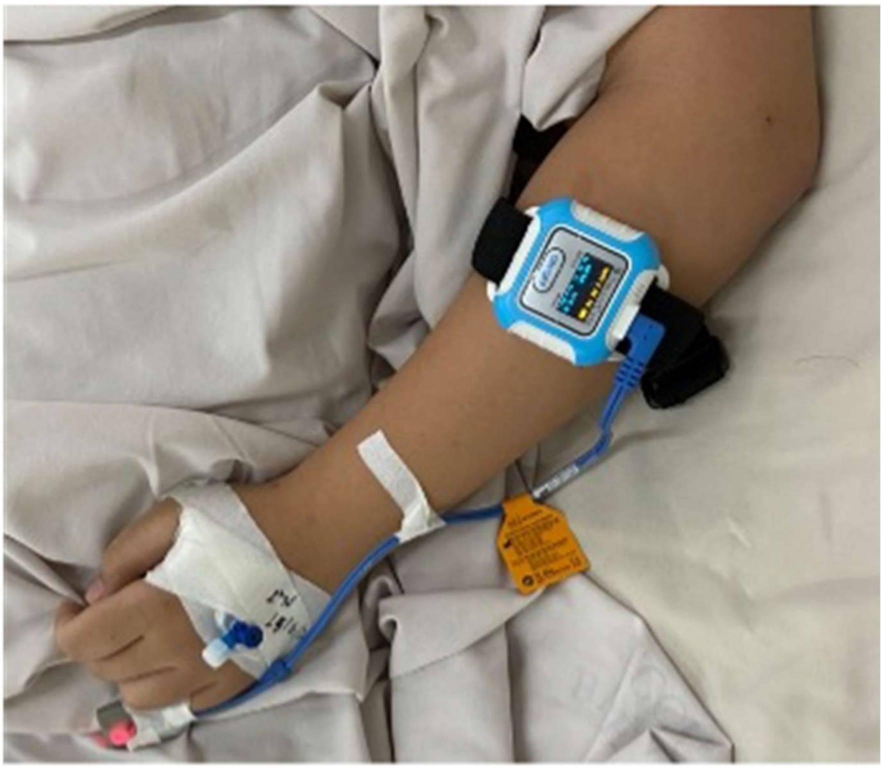

Supplementary figure 2: The distribution of the NEWS2 and mSOFA score (top), and a correlation heatmap (bottom) of the two scores for the analysis cohort (n=153).

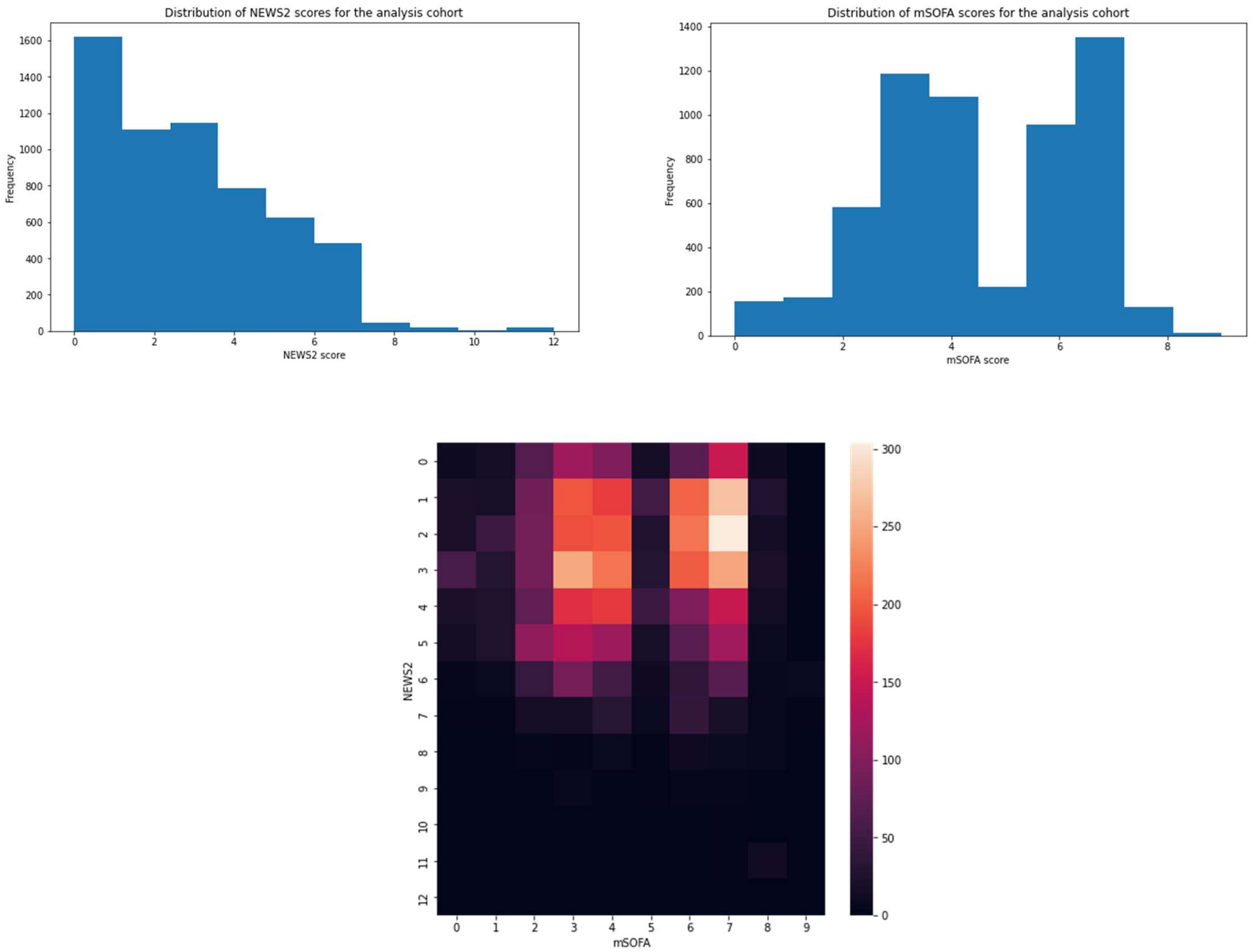

Supplementary table 1: Baseline characteristics of the enrolled cohort of which n=153 patients were included because of data quality or missing data reasons.

		Overall	Excluded	Included
n		234	81	153
age, median [Q1,Q3]		25.0 [16.0,33.0]	13.0 [10.0,22.0]	29.0 [21.0,35.0]
gender, median (%)	Female	131 (56.0)	42 (51.9)	89 (58.2)
	Male	103 (44.0)	39 (48.1)	64 (41.8)
weight (kg), median [Q1,Q3]		56.0 [47.0,68.0]	47.0 [38.8,56.8]	60.0 [52.0,72.0]
pulse (bpm), median [Q1,Q3]		87.0 [80.0,100.0]	86.0 [80.0,99.0]	88.0 [80.0,100.0]
sbp (mmHg), median [Q1,Q3]		110.0 [100.0,120.0]	109.0 [100.0,120.0]	110.0 [100.0,120.0]
dbp (mmHg), median [Q1,Q3]		70.0 [68.0,80.0]	70.0 [60.0,80.0]	70.0 [70.0,80.0]
Respiratory Rate (per minute) median [Q1,Q3]		20.0 [20.0,24.0]	22.0 [20.0,24.0]	20.0 [20.0,22.0]
Body temperature (celsius), median [Q1,Q3]		37.0 [37.0,37.5]	37.0 [37.0,37.5]	37.0 [37.0,37.5]
Haematocrit percent, median [Q1,Q3]		44.4 [40.7,48.0]	43.3 [40.6,47.1]	44.8 [40.8,48.6]
NEWS2_bins, n (%)	1-4	4659 (89.1)	1403 (83.7)	3256 (91.7)
	5-6	335 (6.4)	188 (11.2)	147 (4.1)
	7+	234 (4.5)	86 (5.1)	148 (4.2)
mSOFA_bins, n (%)	1-6	3245 (57.0)	1555 (79.1)	1690 (45.3)
	6-7	2306 (40.5)	393 (20.0)	1913 (51.3)
	8+	143 (2.5)	19 (1.0)	124 (3.3)
clinical_score, n (%)	0	4096 (70.0)	1160 (57.3)	2936 (76.8)
	1	1754 (30.0)	865 (42.7)	8893.2)

Supplementary figure 3: NEWS2 (Royal College of Physicians) scoring system

Chart 1: The NEWS scoring system

Physiological parameter	Score 3	2	1	0	1	2	3
Respiration rate (per minute)	≤8		9–11	12–20		21–24	≥25
SpO_2 Scale 1 (%)	≤91	92–93	94–95	≥96			
SpO_2 Scale 2 (%)	≤83	84–85	86–87	88–92 ≥93 on air	93–94 on oxygen	95–96 on oxygen	≥97 on oxygen
Air or oxygen?		Oxygen		Air			
Systolic blood pressure (mmHg)	≤90	91–100	101–110	111–219			≥220
Pulse (per minute)	≤40		41–50	51–90	91–110	111–130	≥131
Consciousness				Alert			CVPU
Temperature (°C)	≤35.0		35.1–36.0	36.1–38.0	38.1–39.0	≥39.1	

Supplementary table 2: mSOFA score from McBride et al., 2022

System	Score				
	0	1	2	3	4
Respiratory system(*)					
SpO_2/FiO_2, mmHg	≥ 400	< 400	< 315	< 235 and/or respiratory support	< 150 with respiratory support
Coagulation					
Platelets, /µL	≥ 150,000	< 150,000	< 100,000	< 50,000	< 20,000
Liver function					
Bilirubin, mg/dl [µmol/l]	< 1.2 [20]	1.2–1.9 [20–32]	2.0–5.9 [33–101]	6.0–11.9 [102–204]	> 12.0 [204]
Cardiovascular system					
MAP/**PP** and adrenergic agents	MAP ≥ 70 mmHg & **PP ≥ 20 mmHg**	MAP < 70 mmHg or **PP < 20 mmHg**	**PP < 10 mmHg** OR dopamine ≤ 5 µg/kg/min or dobutamine (any dose)	Dopamine 5.1-15 µg/kg/min OR epinephrine ≤ 0.1 µg/kg/min OR norepinephrine ≤ 0.1 µg/kg/min	Dopamine > 15 µg/kg/min OR epinephrine > 0.1 µg/kg/min OR norephinephrine > 0.1 µg/kg/min
Central nervous system					
Glasgow Coma Scale score	15	13–14	10–12	6–9	< 6
Renal function					

Creatinine, mg/dL	< 1.2	1.2–1.9	2.0–3.4	3.5–4.9	> 5.0
Urine output, mL/d				< 500	< 200

Author Information

The members of the Vietnam ICU Translational Applications Laboratory (VITAL) group are as follows:

Oxford University Clinical Research Unit, Ho Chi Minh City, Vietnam:

1. An Phuoc Luu
2. Chanh Quang Ho
3. Duc Hong Du
4. Duc Minh Tran
5. Dung Thi Phuong Nguyen
6. Giang Thi Nguyen
7. Hai Bich Ho
8. Hien Van Ho
9. Hung Manh Trinh
10. Huy Quang Nguyen
11. Khanh Nguyen Quoc Phan
12. Khoa Dinh Van Le
13. Kien Trung Dang
14. Lam Khanh Phung
15. Lieu Thi Pham
16. Ngoc Thanh Nguyen
17. Nhat Tran Huy Phung
18. Phuong Thanh Le
19. Quyen Than Ha Nguyen
20. Thanh Thi Le Nguyen
21. Thy Bui Xuan Doan
22. Trieu Trung Huynh
23. Trinh Huu Khanh Dong
24. Van Minh Tu Hoang
25. Van Thi Thanh Ninh
26. Vuong Lam Nguyen
27. Yen Minh Lam
28. Sayem Ahmed
29. Joseph Donovan
30. Ronald Geskus

31. Evelyne Kestelyn
32. Angela Mcbride
33. Guy Thwaites
34. Louise Thwaites
35. Hugo Turner
36. Jennifer Ilo Van Nuil
37. Sophie Yacoub

Hospital for Tropical Diseases, Ho Chi Minh City, Vietnam:

1. Tam Thi Cao
2. Thuy Bich Duong
3. Duong Thi Hai Ha
4. Nghia Dang Trung Ha
5. Chau Buu Le
6. Thu Ngoc Minh Le
7. Thao Thi Mai Le
8. Tai Thi Hue Luong
9. Phu Hoan Nguyen
10. Viet Quoc Nguyen
11. Nguyen Thanh Nguyen
12. Phong Thanh Nguyen
13. Anh Thi Kim Nguyen
14. Hao Van Nguyen
15. Duoc Van Thanh Nguyen
16. Chau Van Vinh Nguyen

17. Oanh Kieu Nguyet Pham
18. Van Thi Hong Phan
19. Qui Tu Phan
20. Tho Vinh Phan
21. Thao Thi Phuong Truong

University of Oxford, Oxford, United Kingdom:

1. David Clifton
2. Mike English
3. Shadi Ghiasi
4. Heloise Greeff
5. Jannis Hagenah
6. Ping Lu
7. Jacob McKnight
8. Chris Paton
9. Tingting Zhu.

Imperial College London, London, United Kingdom:

1. Pantelis Georgiou
2. Bernard Hernandez
3. Kerri Hill-Cawthorne
4. Alison Holmes
5. Stefan Karolcik
6. Damien Ming
7. Nicolas Moser
8. Jesus Rodriguez Manzano.

King's College London, London, United Kingdom:

1. Alberto Gomez
2. Hamideh Kerdegari
3. Marc Modat
4. Reza Razavi.

ETH Zurich, Zurich, Switzerland:

1. Abhilash Guru Dutt
2. Walter Karlen

3. Michaela Verling
4. Elias Wicki.

The University of Melbourne, Melbourne, Australia:

1. Linda Denehy
2. Thomas Rollinson
